# Lunacy Legislation

**Published:** 1858-10-01

**Authors:** Forbes Winslow


THE JOURNAL

OF

PSYCHOLOGICAL MEDICINE

AND

MENTAL PATHOLOGY.

OCTOBER 1, 1858.

### Art. I.—LUNACY LEGISLATION.

BY FORBES WINSLOW, M.D., D.C.L.

Being the Valedictory Address at the Meeting of the Association of Medical Officers of Asylums for the Insane at Edinburgh. July, 1858.

Before proceeding to the consideration of the special subject of my address, and resigning into the hands of my illustrious successor the distinguished office with which you honoured me when we met in 1857, at Derby, I would beg to congratulate you on our holding this year our usual annual meeting in this justly renowned and classical city.

I cannot conceal from you the great pleasure and gratification which I feel individually, and in which I am sure you all participate, in having this opportunity of meeting on their own native soil not only the members of this, but of the British Medical Association residing north of the Tweed, but of having the privilege of renewing acquaintance with the many distinguished and honoured members of our profession living in this illustrious and far-famed seat of learning.

Gentlemen, we tread on sacred and hallowed ground; we breathe an atmosphere redolent of sweet poetry and of wild, fascinating romance; we hold sweet communion and holy converse with art, literature, and science, in their most exalted types and noblest forms. A halo of genius gilds and encircles every object upon which we fix our attention; associations of the most touching and thrilling character gush upon the mind wherever we turn our steps; we are in the cradle of genius, the emporium of science, the great school of art, the Academic groves of literature and philosophy—

"Hail, Caledonia, name for ever dear,
Before whose sons we're honoured to appear;
Where every science, every noble art,

That can inform the mind or move the heart
Is known . . . . . . . . . .
Philosophy, no idle pedant's dream,
Here holds her search, by Heaven-taught Reason's beam."

Need I say more to awaken in your minds the warmest enthusiasm, than to remind you of the fact that we are in the country rendered immortal by the resplendent genius of a Burns and a Walter Scott? How often have we confessed ourselves spellbound by the magic wand of these great and illustrious poets! How much of our fondest hopes, our boyish affections, our yearnings after the good and the beautiful, our aspirations for worldly fame and distinction, are closely interwoven with the wonderful creations of these mighty gifted men! How the mention of their names brings back to the heart the earliest of our most pleasing, and perhaps sad and painful associations! As we thread our way through this city of enchantment, and gaze on our right and on our left, to the

"*Distant hills,*

*From hidden summits fed with rills,*"

to those many sacred spots enshrined by genius, the memories of the glories of the past rush back to the heart with all the freshness of an early spring. Bidding adieu to the realms of sweet poesy and romance, and considering graver matters of contemplation, the renowned names of Robert Bruce and William Wallace suggest themselves instinctively to our minds. Great, brave, illustrious, and generous warriors, we now pay homage to your virtues and your valour, and worship at your shrine!

Turning from the contemplation of martial glory to the more unostentatious cultivators of science and philosophy, we find in the annals of their great country the names of men pre-eminently distinguished as mathematicians, natural philosophers, logicians, statesmen, geologists, metaphysicians, historians, and jurists, both among the professorships and students of the University.

I will take the liberty of referring to a few of the more distinguished men who have either held office in the University of Edinburgh, or have been educated within its walls. Among the renowned students and graduates of the University of Edinburgh connected with our own profession are the following:—Dr. John Brown, founder of the Brunonian system of medicine; Dr. George Cheyne; William Hunter, brother of the late John Hunter; Sir John Pringle; Dr. James Abercrombie; Sir Gilbert Blane; Dr. Andrew Combe; and Dr. John Reid. Among the celebrated professors, we find the names of the great Cullen, author of the "First Lines;" the two Duncans, one Professor of Mathematics, and the second of the Institutes of Medicine; Dr.

Hope, Chemistry; Sir R. N. Sibbald, Practice of Physic; the two Hamiltons, Alex. and James; Dr. Plummer; John Thompson, Professor of Military Surgery; Dr. James Gregory, Professor of the Practice of Physic; the Monros, *primus, secundus,* and *tertius;* Dr. Black, immortalized by his discoveries of latent heat; Sir Charles Bell; and Robert Liston. The latter, although not officially connected with the University, was a distinguished surgical lecturer in this city. In the list of eminent and illustrious men who were educated in this University, I find the names of Dr. Hugh Blair; James Boswell, immortalized by his "Life of Johnson;" Sir W. Scott; Professor Wilson, known to the public as Christopher North, who was Professor of Moral Philosophy; and Francis, afterwards Lord Jeffrey. Among the celebrated historians are David Hume; Henry; Robertson, made famous by his celebrated "History of America and Scotland," as well as the "Life and Times of Charles the Fifth of Spain;" and Sir Jas. Mackintosh, author of the "Political History of the Last Century." Among the metaphysicians were Reid, Drs. Thos. Brown, Dugald Stewart, and Sir W. Hamilton. In divinity, Dr. Chalmers stands prominently forward; and as a celebrated jurist the name of Erskine, the author of the "Institutes," is entitled to our profound respect. Among the eminent natural philosophers, statesmen, and others distinguished in literature, the following honoured names may be mentioned:—Adam Fergusson, Professor of Natural and Moral Philosophy, and author of the "History of the Roman Republics;" Lord Kames; Alex. Murray, Professor of Hebrew, and well known as the author of the "Philosophic History of the European Languages," and Lecturer on Oriental Literature in this city; John Playfair, Professor of Natural Philosophy and Mathematics; Hutton, the celebrated Geologist; McLaurin, the great mathematician; David Gregory, Professor of Mathematics; and Sidney Smith, Professor of Moral Philosophy. Among the distinguished statesmen who were educated at this University, are Lord Lyndhurst, Lord Campbell, Lord Palmerston, and Lord John Russell. If I were entitled to allude to illustrious living men educated in this great school of medicine and the collateral sciences, I could easily cite a number of names familiar to us as household words and of European reputation.

I do not consider that we sufficiently appreciate in England the immense influence which the Edinburgh, and in fact the whole Scotch, schools of medicine have exercised over the destinies of our profession.

Until the establishment within the last thirty years of three or four great seminaries of medical instruction in England, we were almost entirely indebted to Scotland for the medical education of youth. Nearly all the celebrated English physicians, and many of our distinguished surgeons, undoubtedly acquired in this city and in this country the knowledge which enabled them afterwards to occupy positions of great eminence in our own country.

Let us admit with gratitude the benefit which the Scotch schools of medicine have conferred upon our noble science. Let us never forget how much of the respect in which our professional body is held in all parts of the civilized globe, is mainly owing to the flood of light which has emanated from the men whose genius has made renowned and celebrated this great school of medicine. Can we ever forget the names of the eminent medical luminaries to whom I have called your attention? The recollection of these justly-celebrated men will cluster about our memories as long as the mind retains the power of reviving mental impressions.

The great and illustrious never die. Death has no power and dominion over the genius of man: it is made of imperishable materials. The soul bids defiance to dissolution, and refuses to yield submission to the laws regulating physical decay. Can the great, the good, the intellectual, the noble, ever cease to exist? Can such—

> " . . . . be left forgotten in the dust;
> When Fate, relenting, lets the flowers revive,
> Shall Nature's voice, to man alone unjust,
> Bid him, though deemed to perish, hope to live?
> Is it for this fair Virtue oft must strive
> With disappointment, penury, and pain?
> No! Heaven's immortal spring shall yet arrive,
> And man's majestic beauty bloom again,
> Bright through th' eternal year of love's triumphant reign."

It is impossible to exaggerate and over-estimate the influence which this city has exercised over the moral, political, and social progress and character of the British nation. It has always been considered as the great centre and fountain-head of modern refinement and civilization.

The men who have been educated in the University of Edinburgh, whose minds have been formed and disciplined in this school for the great battle of life, have in many instances risen to the highest positions at the bar and in the senate. It was in this city that Henry Brougham, in conjunction with Sidney Smith, Jeffrey, and Leonard Horner, conceived the idea of the *Edinburgh Review*, a journal which may be considered to have led to the establishment of our own national *Quarterly Review*, and which has always advocated liberal enlightened opinions in literature, art, science, and politics—exercising, consequently, a great influence upon the political, social, and literary relations of our own country.

Independently of the pleasure which we all feel in having this opportunity of enjoying social communion with our medical friends residing in this country, I think we have, altogether apart from such enjoyable considerations, an additional source of congratulation at being enabled at this time to assemble in this portion of the United Kingdom.

The legislative settlement of the long-agitated question of medical reform is a significant fact in the history of modern medicine. It constitutes beyond all question one of the most important epochs within the memory of living medical men. Should not this be a subject for our mutual rejoicing and congratulation?

In the address which I had the honour of delivering in London at our annual meeting last year, I dwelt at some length on the grave responsibilities attaching to those delegated by the Legislature with the legal custody, care, and treatment of the insane.

It is not my intention now to revert to this subject.

Dismissing this topic, I propose to submit to your consideration and for your approval certain suggestions that have occurred to my mind relative to a modification of the laws regulating the care and treatment of the insane.

Mr. Tite, the member for Bath, having in a short speech during the present session of Parliament, brought under the notice of the House of Commons the existing state of Chancery lunatics, with a view to the appointment of a committee to inquire into their condition; and having, on the suggestion of Government, withdrawn his notice of motion, on the promise that in the ensuing session the whole state of the law affecting the care and treatment of the insane would be made the subject of strict Parliamentary inquiry, and, if necessary, legislative interference, I do not think I can more usefully occupy the time of the Association than by calling its attention to what I conceive to be the right basis upon which all legislation regarding the insane should rest. This will open the question for the careful consideration of the Association, and will, I hope, justify the organization of an acting, and of an *active*, committee in London, to watch the progress of future legislation on this important subject—one of all others in which we are personally and collectively deeply interested.

In all legislation relating to the insane it is most desirable that certain first principles regarding the nature of insanity should be freely and fully recognised. In the preamble to every legislative enactment referring to the care and treatment of the insane, the various phases and types of mental disease should be viewed as the effects of certain deviations from a healthy and normal condition of the brain, the great nervous centre, curable by a well-directed course of moral, medical, and hygienic treatment.

Asylums, whether public or private, licensed or unlicensed, whether for one or more patients, ought to be considered as hospitals for the treatment of *a form of brain disorder*, only to be successfully grappled with by educated and experienced medical men.

Much of the mysticism, the superstition, the illusion, and popular fallacy, still unhappily enshrouding the subject of insanity, arises from an indisposition to liberally acknowledge this great and essential first principle.

In my former address I made some allusions to the provisions of the Lunacy Act now in operation; I called the attention of the Association to one of its clauses relating to the unjust exclusion from the office of Commissioner in Lunacy of all who were not in a position to affirm that they had within the previous two years ceased to have any interest in the confinement of persons alleged to be insane. By this clause every member of the medical profession connected with and having an interest, be it ever so infinitesimal, in any private asylum, is virtually excluded from holding office at the Board in Whitehall-place, not having undergone two years' quarantine, and not being in a position to present to the Lord Chancellor (in whose hands are vested the patronage of the office) a clean bill of health,—such being the qualification required by the English legislative enactment for the post of one of her Majesty's Commissioners in Lunacy.

This casual reference to what I had the honour of alluding in my former address brings me at once to the consideration of the constitution of the Board of Lunacy Commissioners, and opens the question, whether it is so organized as to fully meet the requirements of the Act of Parliament, and so selected as to guarantee for it the public and professional confidence? It is not my intention to criticise the proceedings of the present Board of English Commissioners in Lunacy. Like most public bodies delegated by an Act of Parliament with extraordinary power, they may not in every instance keep strictly within their legitimate boundaries. They may occasionally appear to act in an arbitrary and unjust spirit. Nevertheless, I believe they are most anxious to discharge conscientiously their important functions. Considering the extent of their jurisdiction, and the number of insane entrusted to their legal protection, it is a question whether it is not desirable either to add to the number of the existing Board of Commissioners, or to appoint in association with them a body of sub-inspectors or commissioners, with the view of carrying out a more perfect supervision of the insane, in public as well as in private asylums. In any future legislation on the subject of Lunacy, I think it would be desirable to define with more precision the precise legal powers of the Commissioners; and in all cases of serious dispute between the proprietor of an asylum and the Commissioners of Lunacy, I consider it but an act of justice that he should, if he desired it, have the advantage of legal assistance when summoned to appear before the Board to answer any charge which the Commissioners may consider it their duty to bring against him.

Having made these few observations respecting the constitution of the Board of Commissioners, I proceed in the next place to a consideration of the provisions of the existing Act of Parliament relative to the preliminary measures required previously to the confinement of persons alleged to be of unsound mind. I refer to the medical certificates which are imperative in order to justify any kind of restraint, medical or general supervision, on the ground of insanity.

We cannot disguise from ourselves the fact that this part of the Lunacy Act is far from being in a satisfactory condition. There always has been a great outcry against the power which the law places in the hands of two qualified medical practitioners. *Primâ facie* there are undoubtedly grave objections to this clause. If we always could guarantee the respectability, the intelligence, and the practical experience of the members of the medical profession called upon to certify to the mental condition of a person prior to his being placed under legal restraint, no possible objection could be raised to the law as it at present exists; but unfortunately it does occasionally happen that very incompetent men are called in to certify, and by doing so without sufficient ground or reason, serious odium is brought upon all persons associated with asylums for the treatment of the insane. The force of public opinion is beyond all doubt against this part of the legislative enactment; and we had better therefore, with a good grace, bow with submission to the *vox populi*, and consent in this particular to some modification of the law.

It is urged by those who object to the power delegated by the act to two qualified medical practitioners, that it is not right, because the insane are no longer treated with great harshness, cruelty, and brutality, not being chained, whipped, and tortured as they were in the barbarous times long since passed away, that therefore any man should be subjected to restraint in an asylum conducted by men of unquestionable respectability, and in conformity with the most enlightened and humane principles of treatment, on the simple written testimony of two apothecaries who may never have had any previous experience in the investigation of cases of imputed insanity. I consider it to be our bounden, our sacred duty, by some amendment of the law, to fully satisfy the public mind that every safe and proper precaution has been taken to thoroughly examine, in the minutest particular, the mental condition of every person represented to be of unsound mind, and a fit subject to be deprived for a time of his civil rights.

From what I know of the existing state of public feeling upon this point, I feel assured that it would be unwise on the part of those personally interested in the confinement of the insane to offer any opposition to an amendment of the law relative to medical certificates. It has been proposed, with a view of obviating this difficulty, and bringing the Act of Parliament more in harmony with the force of public opinion, that a *quasi*-judicial investigation should be instituted in every case previously to confinement. The Law Amendment Society suggested that an inquiry, similar to a commission of lunacy, should take place prior to the exercise of restraint, and advised that no person should be removed to a lunatic asylum who had not been pronounced by a competent jury to be of unsound mind, and in a condition to justify this mode of treatment. I am sure I need not occupy your valuable time in pointing out the absurdity and impracticability of this suggestion. With a view to the constitution of a less exceptional and incorruptible tribunal, it is proposed that a *Court of Commissioners of Insanity* should be formed, consisting of six or seven experienced men of high repute, who should be empowered to decided on the necessity of restraint in every case of alleged insanity. This Court is to be delegated with the authority of examining medical men upon oath, and if necessary, seeing the person presumed to be insane. Were such a preliminary course necessary in order legally to confine the insane, I very much fear it would greatly add to the statistics of chronic and incurable insanity. I think it would be most unwise, injudicious, and impolitic to throw any very stringent or vexatious impediments or obstructions in the way of confining the insane. Sensible, as all must be who practise in this department of medicine, of the enormous curative advantages which result from the immediate removal of cases of acute insanity from the associations of home to a well-organized and humanely-conducted institution for the treatment of morbid conditions of mind, it behoves us to sanction no alteration in the law that would obviously and seriously interfere with this important principle of treatment. What is the alteration, it may be asked, that I would suggest to meet the difficulties referred to? The law at present requires that two qualified medical men should personally, and apart from any other practitioner, examine the patient, and certify to the fact of insanity, specifying at the same time the facts upon which they have based their opinion.

It has been proposed, with a view to altering the law and satisfying the requirements of public opinion, that instead of two medical certificates, *three*, or even *four*, should be required in every case previously to the imposition of restraint, and that at least *one* or two of the certificates should bear the signatures of physicians of high character and of known repute and experience. I am bound, however, to confess, from what I know of the state of public feeling on this point, that even this great concession to the popular outcry would not be satisfactory. To meet the objections raised, and to place this matter beyond all further cavil and dispute, I would suggest the appointment of educated, respectable, and experienced practitioners, delegated with *quasi*-judicial and magisterial functions, to be summoned for the purpose of counter-signing the certificates of the medical men, thus sanctioning, if they thought proper, the proposed measure of confinement. These Inspectors of Lunacy, or medico-legal jurists, might be appointed to preside over certain districts in the metropolis as well as in the provinces. Being unconnected with and unknown to the relations and friends of the patient, and strictly independent of the medical men called in by the family to certify to the fact of insanity, I feel assured that the signatures of gentlemen holding such independent official appointments would relieve the public mind of all undue anxiety relative to the unjust confinement of persons alleged to be insane. I think, also, it would be considered as a boon to the medical men certifying to the fact of insanity, as well as to the family of the invalid, by placing their conduct in the matter beyond all doubt and suspicion.

There are one or two other points in connexion with the medical certificates to which I would beg to call the attention of the Association. Having dwelt upon the importance of adopting efficient measures of protecting the alleged lunatic from unjust confinement and detention in an asylum, I would suggest that in some cases of mental disorder, and mental disorder of such a kind and degree as to justify residence in an asylum or private house, the certificate of insanity should, under specific and peculiar circumstances, be altogether dispensed with.

In the existing state of the law, no person alleged to be of unsound mind can be placed under medical, moral, or general supervision in an asylum, or in a private house or lodgings (the party keeping such house or lodgings receiving payment for the board or maintenance of such patient), without two certificates of insanity. The Act of Parliament makes it also imperative on the part of the person admitting such patient into his private dwelling, to make an official return to the Commissioners in Lunacy of the fact, accompanying such representation with a copy of the certificates and order upon which he was admitted.

In common with many medical men engaged in the treatment of the insane, I viewed this provision of the Lunacy Act as an obvious and important improvement upon the previously existing statute. I consider, however, it now to be my duty to state that I have seen good reasons for modifying my opinion of this section of the Act of Parliament. I think the law with regard to the confinement of persons in private lodgings and in unlicensed houses is too stringent in its operation.

There is a vast amount of incipient insanity and morbid conditions of mind connected with obscure brain disease, that require, with a view to the adoption of efficient medical curative treatment, to be removed temporarily from irritation and excitement, often necessarily incidental to a continuance among relations and friends. In many of these cases no progress towards recovery can be made until the patient is removed from home, and ceases for a time to be a free agent. Under kind and skilful treatment these patients rapidly recover; but in order to effect so desirable a consummation it is essential that they should be placed among strangers and under judicious control. Is it not unwise, I would ask, that the law should make it imperative that this class of mental invalids should be formally certified to be insane, and registered as such at the office of the Commissioners in Lunacy? The fact of a patient being placed under temporary restraint whilst suffering from an attack of transient mental aberration, does not at all affect his social position should he recover and return home to his family, but the position of this patient would be materially altered if he had been certified to have been insane, and visited as such by the gentlemen appointed by the Act of Parliament to examine all persons legally confined as lunatics. I am quite satisfied that there are many patients who are kept at home under great disadvantages, as far as the question of recovery is concerned, in consequence of this stringent provision of the law.

So great is the horror which some sensitive persons exhibit at the bare mention of a certificate of lunacy, that they have confessed a determination, rather than submit to what they conceive to be a seriously damaging stigma, to abandon all idea of bringing those near and dear to them within the range of remedial measures.

Could not some modification of the law be suggested to meet this class of case? Would not the public be sufficiently protected from the interference of their friends and relations, if every person admitting such uncertified cases into his house or lodgings were compelled to make a return of the fact to the proper authorities, viz.: the Commissioners in Lunacy, or the district medical inspector, medical jurist, officer of health, or by what other name it may be thought proper to designate these official personages?

There are numerous cases that require, for their own safety as well as the security and happiness of others, to be sent from home in consequence of some apparently trifling mental infirmity. It is often essentially requisite that such persons should be placed under the control and supervision of strangers. In this type of case no kind of justification can be urged for having them certified as lunatics. Again, I would suggest an alteration in the certificates required for the admission of private patients into licensed establishments for the treatment of the insane.

It has often occurred to me, and I have no doubt to all officially associated with private asylums for patients conscious of their mental disorder, fully recognising the loss of self-control, bitterly bewailing being the prey to morbid impulses, to express a wish to be placed under restraint. I have known patients to drive up to the door of the asylum and beg to be received within its walls, being painfully and acutely conscious of the necessity of close supervision. Great have been the lamentations when they have been informed that they could not be admitted even for one night into the asylum, without being certified by two medical men to be in an insane state of mind. I have known such persons take the printed form of admission, and go themselves to medical men in the neighbourhood, and beg them to sign the legal certificates of insanity. Why should there not be some alteration of the legislative enactment to remedy this defect? If a person recognising his morbid condition of mind, and anxious to subject his case to medical treatment, voluntarily offers to surrender his free agency into the hands of the medical head of a lunatic asylum, the law should not force him against his will to be formally certified and registered as a lunatic. In such cases I would compel the patient to sign, in the presence of a justice of the peace or magistrate, a paper, to the effect that he, in consequence of mental indisposition, freely, voluntarily, and without compulsion, places himself in a licensed asylum for the treatment of the insane. A copy of this document, with all the particulars of the case, should be transmitted to the Commissioners of Lunacy within a few hours of admission.

If it were thought desirable for the protection of the public that these patients should go to the Commissioners themselves, and obtain their authority for entering the asylum as patients uncertified to be insane, no possible objection can be made to this course of procedure. In all legislation on the subject of lunacy, it is most important to studiously avoid throwing any vexatious impediments in the way of bringing the insane as speedily as possible within the reach of curative agents.

A full and liberal recognition of this great principle of treatment is quite consistent with the adoption of very stringent means for the protection of the public against all unjust interference and confinement on the ground of insanity.

It was my intention before concluding this address, to have called the attention of the Association to some other suggestions that have occurred to my mind relative to the state of the lunacy laws, not restricting my remarks to the Act of Parliament which takes special cognizance of the insane subject to restraint in licensed and unlicensed houses. I was anxious to make some remarks respecting the defective state of the law bearing upon cases of alleged mental unsoundness and incapacity which so often come before our courts of law in the form of commissions of lunacy.

I am of opinion that the law relating to these cases requires careful revision.

At present, no condition of mental incapacity is recognised by the jurists of this country apart from actual unsoundness of mind in its legal signification; and such a condition of the intellect must be established by evidence before the Court of Chancery will appoint a guardian or a committee to administer to and protect the property of the person alleged to be of unsound mind, and thereby incapable of managing his own affairs.

The writ *de lunatico* directed by the Court of Chancery to the Masters in Lunacy, authorizes these judicial functionaries to inquire into the *insanity, idiotcy,* or *lunacy* of A or B; and no type of case can be legally dealt with by the Master which is not embraced within one of these three divisions. It is true that the modified and less offensive phrase, "unsoundness of mind" (which never yet has been satisfactorily defined by lawyers or physicians), is adopted during the proceedings preliminary to the issuing of the writ *de lunatico,* and at the time of the judicial inquiry; but if the party be declared to be of "unsound mind," either by the Master or by the jury, he is in all the subsequent proceedings designated as a "chancery *lunatic,*" and in the eye of the law he is so considered, should he not recover, until the day of his death!

But may not a person be quite incompetent to take care of himself and manage his property, without being either insane or a lunatic? and would it not be a gross, unjustifiable, and cruel misapplication and perversion of language, so to consider and designate those who, either from cerebral disease, an accidental cause, or premature decay of intellect, are reduced to this sad condition of physical and mental helplessness? There is a vast body of persons in this state of infirm and enfeebled mind who are entitled to and who should have extended towards them legal protection.

Men in this state of *quasi*-insanity contract foolish and improvident marriages ; are facile in the hands of designing domestics and unprincipled knaves ; they are persuaded to squander recklessly their property; large sums are often exacted from them ; they are induced to make testamentary dispositions adverse to the claims of relationship and the ties of consanguinity, and in conformity with the wishes and interests of those who have obtained improper and undue influence over the poor broken-down and impaired intellect.

To meet the exigencies of this numerous class of cases, there should be some short, summary, inexpensive mode of legal procedure, quite distinct in its character from ordinary commissions of lunacy. Persons so enfeebled in mind as to be palpably unfit for the management of their property, might be placed under the guardianship or tutorship of one or two members of the family, by some simple judicial process, without rendering it necessary that they should be formally declared to be of unsound mind by the judge, and registered in the records of the Court of Chancery as lunatics.

Protect the property and persons of these unhappy individuals by the most stringent means that can be devised and concocted, but save them and their families from the social disadvantages that would result from their being declared to be insane!

I am satisfied that an alteration in the law similar to that I have suggested is imperatively demanded, and would, if carried into effect, be productive of a vast amount of good to the community.

I fear, gentlemen, I have exhausted your patience. I had much more to say to you on this important subject, but at present I shall not further trespass upon your valuable time. Thanking you most sincerely for the confidence which you have so generously extended towards me during my period of office, I now resign my post into the hands of one much more competent to discharge its duties than the humble individual who now has the honour of addressing you.

